# Supplementary material for: Can internal medicine specialists diagnose functional somatic disorders (FSDs)? Training and comparison with FSD specialists
Source: PLoS One. 2024 Jul 12;19(7):e0307057. doi: 10.1371/journal.pone.0307057 (PMC11244839; doi:10.1371/journal.pone.0307057)
Supplement: Attachment — Submitted filename: Reveiw report PLOS ONE.docx [file pone.0307057.s002.docx]

The theme of the topic is good, and the paper provides a thorough literature review and background study. Still, the paper has some hindrances in justifying the study in the introduction section, the importance of selected models in the literature section, and the selected model should be validated in the methods and data section. I suggest the publication, but after considering the following minor comments.

1: Study Design: when was the study conducted?

2. The Introduction Section needs to be improved with relevant and updated references.

3: Time per patient: While the abstract mentions that internists require 3-4 hours per patient, it does not provide information on the practicality or feasibility of this time requirement in real-world clinical settings.

4: Generalizability: The study participants were referred to a non-psychiatric diagnostic clinic, which may limit the generalizability of the findings to other settings or populations.

5: Small sample size: The study included only 27 patients, which may limit the generalizability of the findings. A larger sample size would provide more robust results and increase confidence in the conclusions drawn.

6. Check the presentation and Grammar of the article.

7: Improve the quality of your figure in the manuscript.
