# Supplementary material for: Can internal medicine specialists diagnose functional somatic disorders (FSDs)? Training and comparison with FSD specialists
Source: PLoS One. 2024 Jul 12;19(7):e0307057. doi: 10.1371/journal.pone.0307057 (PMC11244839; doi:10.1371/journal.pone.0307057)
Supplement: Attachment — Submitted filename: Response to Reviewers.docx [file pone.0307057.s003.docx]

Michael Moesmann Madsen

Department of Clinical Medicine - Research clinic for Functional Disorders

Palle Juul-Jensens Boulevard 11

DK-8200 Aarhus N

michael.madsen@rm.dk

Denmark

Dr. Zulqarnain Mushtaq, PhD

Academic Editor

PLOS ONE

[plosone@plos.org](mailto:plosone@plos.org)

Response to Reviewer Comments - PONE-D-23-38611 - "Can Internal Medicine specialists diagnose Functional Somatic Disorders (FSDs)? - Training and comparison with FSD specialists"

Dear Dr. Mushtaq,

We would like to express our sincere gratitude to the reviewers for their constructive comments and to you for the opportunity to improve our manuscript. We have carefully considered each point raised and have made corresponding revisions to the manuscript. Please find below a detailed response to each of the comments:

**Study Design (Comment on dates of the study): *“Study Design: when was the study conducted?”***

The dates are listed in the study in the Methods section lines 122-123 (previous version of the manuscript). However, to clarify and highlight the temporal scope within which our research was conducted, we have also added “The study was conducted between May 2020 and April 2022” to the abstract.

**Introduction Section (Need for updated references): *“The Introduction Section needs to be improved with relevant and updated references.”***

Thank you for your valuable feedback regarding the need for updated references in the Introduction section of our manuscript. In response to your comments, we have added several additional relevant references. If there are specific recent articles you believe should be included to enhance our introduction, please feel free to mention them for our consideration.

**Time per Patient (Practicality and feasibility):** *“Time per patient: While the abstract mentions that internists require 3-4 hours per patient, it does not provide information on the practicality or feasibility of this time requirement in real-world clinical settings.”*

We have added a discussion in the Limitations section (now also reflected in the Introduction) about the practical challenges of implementing extended visit times in the context of to the “same-day diagnostic” approach used in this and other diagnostic centres, where extended consultations can lead to more efficient patient management, albeit requiring adjustments in scheduling and resource allocation. Additionally, the economic impact of this approach is being assessed in the ongoing DISTRESS trial (NCT06025617), providing crucial data on its cost-effectiveness.

**Generalizability (Referral to a non-psychiatric diagnostic clinic): *“The study participants were referred to a non-psychiatric diagnostic clinic, which may limit the generalizability of the findings to other settings or populations.”***

Thank you for your comment regarding the generalizability of our findings. We have adapted the introduction to reflect the fact that patients included in our study are typically managed within general (physical) medicine diagnostic centres. Given that these patients present with physical symptoms and physical diagnostic uncertainty remains, these patients are rarely if ever referred to psychiatric departments. By transferring knowledge from the highly specialized clinic for functional disorders to a general physical (general medicine) diagnostic center, we thus arguably augment the provision of the right level of care in the most appropriate setting, enhancing the relevance and applicability of our findings to similar healthcare systems.

**Small Sample Size (Limitations on generalizability): “*Small sample size: The study included only 27 patients, which may limit the generalizability of the findings. A larger sample size would provide more robust results and increase confidence in the conclusions drawn.”***

Acknowledging the limitation posed by our sample size, we have refined our discussion in the Limitations section. We removed the qualifying parenthesis and emphasized the need for further research with larger sample sizes to enhance the robustness of the findings.

**Presentation and Grammar: *“Check the presentation and Grammar of the article.”***

We have submitted the manuscript for a thorough language review to ensure the clarity and grammatic correctness of the text.

**Quality of Figure: *“Improve the quality of your figure in the manuscript.”***

We have used the PACE preflight system to improve the figure's quality. Do let us know if we need to revise the figure additionally.

Attached, please find the revised manuscript (marked-up and clean versions) and the updated figure as per PLOS ONE's guidelines. We believe that these revisions have substantially improved our manuscript and hope that it is now suitable for publication in PLOS ONE.

Thank you for considering our revised manuscript. We appreciate the chance to make these improvements and look forward to your decision.

Sincerely,

Michael Moesmann Madsen
